# Rural/Urban differences in uptake of preventive healthcare services: Variability in observed relationships across measures of rurality

**DOI:** 10.1177/22799036241238670

**Published:** 2024-03-18

**Authors:** Brittney M Calatayud, Jennifer L Moss

**Affiliations:** Penn State College of Medicine, Hershey, PA, USA

**Keywords:** Rurality, preventive healthcare, colorectal cancer screening, cervical cancer screening, private health insurance

## Abstract

Rural residents are generally less likely to receive preventive healthcare than are urban residents, but variable measurement of rurality introduces inconsistency to these findings. We assessed the relationships between perceived and objective measures of rurality and uptake of preventive healthcare. In our sample, rural participants generally had equal or higher uptake of healthcare (i.e. private health insurance, check-up in the past year, being up-to-date on colorectal and cervical cancer screening) than urban participants. Importantly, the perceived measure of rurality performed similarly to the objective measures, suggesting that participant report could be a valid way to assess rurality in health studies.

Significance for Public Health

The ability to access routine preventive healthcare is a key component of public health. Comparing uptake of cancer screening in rural versus urban areas is one way to assess equity of healthcare access. Generally, rural areas have a higher burden of cancer than urban areas. The built environment, socioeconomic status, and patient perceptions can impact an individual’s access to routine cancer screening. Preventive healthcare is of great importance to public health as a whole because screening can facilitate earlier diagnosis and more successful treatment for many preventable cancers, which may ultimately increase the quality and quantity of life.

Rural/urban differences are apparent in many health conditions, with rural areas generally having higher burden of disease.^1,2^ This pattern may be explained by lower likelihood of obtaining certain preventive healthcare services among rural residents,^3^ which in turn is driven by differences in socioeconomic status, public transportation infrastructure, distances between homes and clinical services, and perceived importance of routine health maintenance.^4,5^

Preventive healthcare services are important for increasing quality and quantity of life. For example, screenings for colorectal or cervical cancer can prevent mortality because they facilitate earlier detection of smaller, more curable tumors.^6^ As a result, the U.S. Preventive Services Task Force (USPSTF) recommends routine cancer screening.^7,8^ Despite this recommendation, rural residents are less likely to receive screening for these cancers than are urban residents.^9,10^

However, there is wide variability across research studies in how rurality is measured,^11,12^ creating confusion about definition, interpretation, and consistency of rural/urban differences in health. Comparing relationships between measures of rurality and uptake of preventive healthcare services could provide insight into which measure is most appropriate for a given research question.

## Methods

### Data source

This study is a secondary analysis of data from a survey examining barriers to cancer screening.^13^ Participants were residents of 28 counties in the Penn State Cancer Institute catchment area; female; 45–65 years old; and able to speak and read in English. Participants were recruited through social media advertisements and, after providing consent, they completed the survey over telephone or online. Participants were compensated for their time with a $15 gift card. Between November, 2019, and May, 2020, 474 participants completed the survey. Additional details on the study methods can be found elsewhere.^13^

In the present analysis, we geocoded self-reported home address, excluding 45 participants (9.5%) who provided an incomplete address, a PO box, or their work address as their primary address. Thus, our analytic sample included 429 participants.

### Measures

#### Rurality measures

We collected several measures of rurality. First, we captured perceived rurality with a single survey item: “From your perspective, is the community where you live. . .” with response options of urban, suburban, or rural.

Second, we categorized rurality based on county-level data using the 2013 rural-urban continuum codes (RUCC) developed by the U.S. Department of Agriculture.^14^ Counties with RUCC of 1–3 are considered metropolitan (or “urban”), and counties with RUCC of 4–9 are considered non-metropolitan (or “rural”). Survey recruitment was designed to achieve equal numbers of participants from rural versus urban counties using RUCC.

Third, we categorized rurality based on two measures using census tract-level data. The first measure was Rural Urban Commuting Areas (RUCAs), which we used to classify census tracts as metropolitan (or “urban”) if they had a RUCA of 1–3 and non-metropolitan (or “rural”) if they had a RUCA of 4–10.^15^

The second measure was Urbanized Areas/Urban Clusters (UA/UC), which we used to classify census tracts as UA/UC (or “urban”) if they were in an urbanized area or cluster, and not UA/UC (or “rural”) if they were outside of an urbanized area.^16^

#### Outcome measures

Self-reported outcome measures captured access to and uptake of preventive healthcare services: healthcare insurance type (private vs other); having a check-up in the last year (yes vs no); and being up to date with colorectal cancer screening (yes vs no) and cervical cancer screening (yes vs no), according to USPSTF guidelines.

### Statistical analysis

First, we generated descriptive statistics to characterize our analytic sample. We calculated mean and standard error (SE) for participant age as well as counts and frequencies for participant race/ethnicity (non-Hispanic White vs other), educational achievement (high school [HS] degree or less vs more than a HS degree), and annual household income (<$50,000 vs $50,000 or more).

Next, we used multivariable logistic regression to assess the relationships between each rurality measures and each outcome measure, controlling for participant age, race/ethnicity, educational achievement, and annual household income. We calculated the odds ratio (OR) and 95% confidence interval (CI) for the relationship between each rurality measure and preventive healthcare. We compared the consistency of these relationships by assessing the direction of the OR and overlap of the CIs.

The Human Research Protection Program at the Pennsylvania State University determined that data collection and analysis for this study was exempt from ongoing review. Data analysis was conducted using SAS version 9.4 (Cary, NC) using a two-sided *p*-value of 0.05.

## Results

The 429 participants had a mean age of 55.1 years (SE = 0.3) (Table 1). Only 13 participants (3%) reported a race/ethnicity besides non-Hispanic White. Most participants had educational achievement beyond a high school degree (87%) and had an annual household income of $50,000 or more (74%). Across the four measures of rurality, 59.7% were rural according to perceived rurality; 49.9% according to county-level RUCC; 40.1% according to census tract-level RUCA; and 37.7% according to census tract-level UA/UC.

**Table 1.** Characteristics of participants (*n* = 429).

	Mean	St. err.
Age, years	55.1	0.28
	*N*	%
Race		
Non-Hispanic white	404	97
Other	13	3
Educational achievement		
HS degree or less	54	13
More than a HS degree	360	87
Annual household income, $		
<$50,000	108	26
$50,000+	303	74

**Figure 1.**
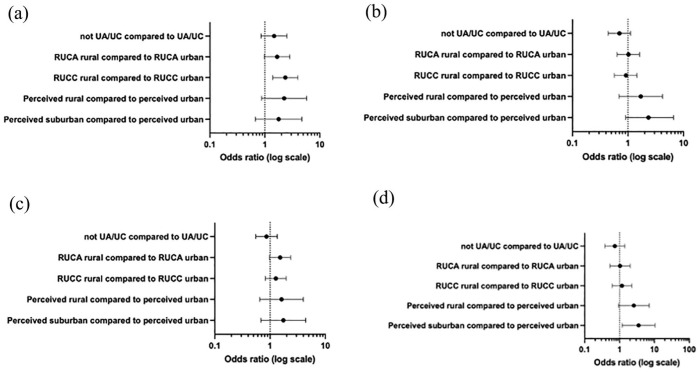
Summary of relationships between rurality measurements and (a) having private health insurance, (b) having a recent health check-up, (c) being up-to-date (UTD) with colorectal cancer screening, and (d) being UTD with cervical cancer screening. RUCA: rural-urban commuting area; RUCC: rural-urban continuum code; UA/UC: urbanized area/urbanized cluster. Error bars indicate 95% confidence intervals.

First, 72.9% of participants had private health insurance. Compared to urban participants, rural participants generally were more likely to have private insurance, although this relationship only achieved statistical significance for the county-level RUCC measure (OR = 2.36, 95% CI = 1.39–4.01) (Figure 1(a)).

Second, 75.0% of participants had a recent health check-up. There was no statistical difference in having a recent check-up for rural compared to urban participants across measures of rurality, and the direction of the relationship between rurality and having a recent check-up differed for perceived rurality and RUCA (i.e. positive) compared to RUCC and UA/UC (i.e. negative) (Figure 1(b)).

Third, 57.3% of participants were up to date with colorectal cancer screening. There was no statistical difference in colorectal cancer screening for rural compared to urban participants across measures of rurality, but the direction of the relationship was generally consistent and positive across measures (Figure 1(c)).

Fourth, 83.2% of participants were up to date with cervical cancer screening. Compared to urban participants, rural participants generally were more likely to be up to date with cervical cancer screening, although this relationship only achieved statistical significance for the suburban-level measure of perceived rurality (OR = 3.52, 95% CI = 1.19–10.39) (Figure 1(d)).

## Discussion

Our analysis aimed to compare the direction and magnitude of the relationship between rurality and access/uptake of preventive healthcare services across rurality measures (Figure 1). The expected outcome, based on findings from previous research,^3–5,9,10^ was that participants from rural communities would have reduced uptake of healthcare compared to participants from urban communities. However, in our sample, rural participants generally had equal or higher access to healthcare than the more urban participants. Specifically, rural participants were more likely to have private insurance (according to county-level RUCC) and to be up-to-date with cervical cancer screening (according to perceived rurality) than urban participants. The direction and magnitude of rural/urban differences were mostly consistent for the remaining (non-statistically significant) relationships, with the exception of census tract-level UA/UC, which often demonstrated poorer access to healthcare for the more rural participants. This overall pattern of equal/higher access among rural versus urban participants may have emerged due to the high socio-economic status of the rural participants in this study compared to the larger rural population in Pennsylvania and the whole U.S.^17,18^

Previous studies have examined the utility of objective county- and census tract-level measures of rurality in explaining health outcomes,^12,19^ concluding that these measures may operate differently depending on the outcome of interest. An important contribution of our study is the comparison of perceived as well as objective indicators of rurality. We found that relationships between rurality and preventive healthcare were similar for the perceived versus objective measures. This similarity suggests that participants can accurately summarize their communities in ways that are meaningful for their preventive healthcare. Therefore, participant-reported perceived rurality could be just as useful for research studies as are objective measures derived from the more arduous process of geocoding, which is an important note for future researchers.

As described above, the UA/UC measure of rurality tended to generate estimates of rural/urban differences in healthcare access that were different in direction from the other measures. Long et al.^12^ previously demonstrated that using the UA/UC measure may result in consistently smaller estimates of rural/urban health disparities, which they attributed to the breadth of this definition and the non-linear relationship between rurality and health. Our results were generally consistent with these conclusions. Additional research is needed to understand how UA/UC differs from other measures of rurality and the implications for studies of health disparities.

### Strengths and limitations

The primary strength of this study was the ability to compare relationships with healthcare for several perceived and objective measures of rurality. Limitations include the low representativeness of the rural sample, which, as noted, generally had higher levels of education and annual income than average for rural Pennsylvania and the U.S.^16,17^ In addition, the sample was primarily non-Hispanic White and limited to female participants between 45 and 65 years of age. More heterogeneous groups may experience different facilitators and barriers to healthcare across levels of rurality. In addition, the outcomes are self-reported, which may be vulnerable to recall and social desirability biases. Data collection was completed in May 2020, that is, toward the beginning of the COVID-19 pandemic; healthcare access has changed markedly since then, with perhaps greater reductions in access in rural communities.

## Conclusions

In conclusion, rurality is related to preventive healthcare, and our analyses demonstrate that the associations between rurality and healthcare access are similar across commonly used measures. Future research should extend these findings to account for healthcare accessibility, health attitudes and perceptions, and barriers to care in rural areas.
